# Negative E-cadherin expression on bone marrow myeloma cell membranes is associated with extramedullary disease

**DOI:** 10.12688/f1000research.109551.2

**Published:** 2022-11-30

**Authors:** Maki Hirao, Kohei Yamazaki, Kentaro Watanabe, Kiyoshi Mukai, Shigemichi Hirose, Makoto Osada, Yuiko Tsukada, Hisako Kunieda, Ryunosuke Denda, Takahide Kikuchi, Hiroki Sugimori, Shinichiro Okamoto, Yutaka Hattori

**Affiliations:** 1 Division of Hematology, Department of Medicine, Saiseikai Central Hospital, 1-4-17 Mita, Minato-ku, Tokyo, 108-0073, Japan; 2 Department of Medicine, Sowa Hospital, 1752 Oshima, Midori-ku, Sagamihara, Kanagawa, 252-0135, Japan; 3 Department of Diagnostic Pathology, Keiyu Hospital, 3-7-3 Minatomirai, Nishi-ku, Yokohama, Kanagawa, 220-8521, Japan; 4 Department of Diagnostic Pathology, Saiseikai Central Hospital, 1-4-17 Mita, Minato-ku, Tokyo, 108-0073, Japan; 5 Department of Hematology, National Cancer Center Hospital, 5-1-1 Tsukiji, Chuo-ku, Tokyo, 104-0045, Japan; 6 Department of Preventive Medicine, Daito Bunka University Faculty of Sports and Health Sciences Graduate School of Sports and Health Sciences, 560 Iwadono, Higashimatsuyama, Saitama, 355-8501, Japan; 7 Division of Clinical Physiology and Therapeutics, Keio University Faculty of Pharmacy Graduate School of Pharmacy, 1-5-30 Shibakoen, Minato-ku, Tokyo, 105-8512, Japan

**Keywords:** E-cadherin, extramedullary disease, multiple myeloma, immunohistochemistry

## Abstract

**Background: **The loss of E-cadherin expression and the induction of N-cadherin are known as hallmarks of the epithelial-to-mesenchymal transition, an essential initial step in the process of metastasis in solid tumors. Although several studies have reported expressions of these cadherins in patients with multiple myeloma (MM), their clinical significance is unknown as MM cells are non-epithelial.

**Methods:** In this study, we examined the expression of E- and N-cadherins by immunohistochemistry using bone marrow (BM) biopsy specimens from 31 newly diagnosed MM patients and in subsequent biopsy specimens from six of these.

**Results:** Negative E-cadherin expression on BM myeloma cell membranes was significantly associated with the presence of soft-tissue masses arising from bone lesions and breaking through the cortical bone, referred to as extramedullary disease (EMD).

**Conclusions:** Given the aggressive nature of EMD, our study suggests that screening for E-cadherin using BM immunohistochemistry is one measure that could predict the development of EMD in patients with MM.

## Introduction

The loss of E-cadherin expression and the subsequent induction of N-cadherin are known as hallmarks of the epithelial-to-mesenchymal transition, an essential initial step in the process of metastasis in solid tumors. ^1^ Although multiple myeloma (MM) cells are non-epithelial cells, Roccaro *et al*. and Azab *et al*. ^2^^,^^3^ suggested that reduced E-cadherin expression in MM cells can promote extramedullary disease (EMD) *in vitro* and in an animal model, partly due to epithelial-to-mesenchymal-transition-like features. EMD is defined as the presence of an MM-cell tumor outside the bone marrow (BM), either in the form of a soft-tissue mass spreading contiguously from the bone and breaking out of the cortical bone (osseous) or arising in an isolated organ not contiguous with bone lesions (extraosseous). ^4^ Epithelial-to-mesenchymal transition-like features in MM patients can be used as biomarkers for EMD; however, the clinical significance of E- and N-cadherin expressions, especially in BM specimens of patients with MM, is still largely unknown and thus needs to be elucidated.

In this article, we examined E- and N-cadherin expression on MM cells obtained from patients using immunohistochemistry. We tried to clarify whether the expression of these cadherins is associated with the presence of EMD and other clinical parameters.

## Methods

### Study group

This study was approved by the ethics committee of Saiseikai Central Hospital (Rin 28-66) and Keio University Faculty of Pharmacy (190605-3). Written informed consent for publication of the patients’ details and their images was obtained from the patients. This retrospective study included 89 consecutive patients (49 men and 40 women) between the ages of 36-94 years old (mean: 70 ± 13 years) between January 2009 and December 2016 who were newly diagnosed as having MM at Saiseikai Central Hospital. All eligible study patients with newly diagnosed MM did not receive any therapy at the time of BM biopsy. Inclusion criteria were provision of full data available on EMD involvement (yes or no) at time of diagnosis, its location, and the number of EMDs. After the exclusion of cases who were lost at follow-up or lacked indispensable medical documents, 31 patients (18 men and 13 women) between the ages of 46-88 years old (mean: 67 ± 11 years) were selected for this study. A total of 31 BM samples were collected at diagnosis and five BM samples and one surgically removed extraosseous EMD sample were collected during disease progression. The median observation period was 46 months (range 3-93).

Disease stage for each patient was based on the International Staging System. ^16^ Serum concentrations of albumin, β2 microglobulin, calcium, creatinine, lactate dehydrogenase and hemoglobin were measured as a part of routine medical care.

### Immunohistochemistry

**Figure 1.**
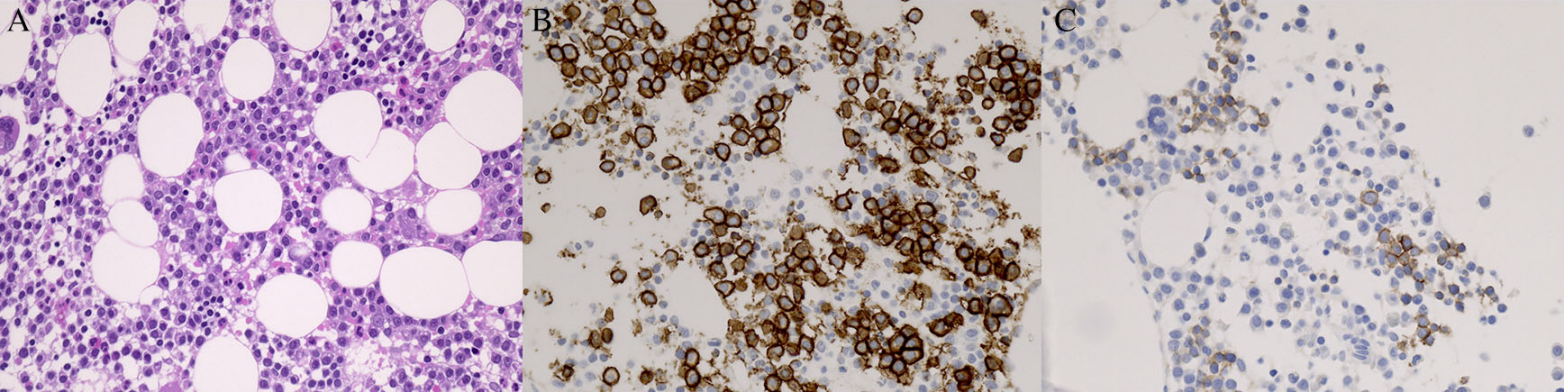
Typical immunophenotype of E-cadherin-positive MM cells (original magnification ×400). (A) BM biopsy with MM cell infiltrate. Hematoxylin and eosin stain. Consecutive sections of BM biopsies were stained with antibodies against (B) CD138 and (C) E-cadherin. Note that E-cadherin was clearly stained in the membrane of MM cells. MM, multiple myeloma; BM, bone marrow.

Immunohistochemistry for E- and N-cadherin was performed on BM and EMD biopsy specimen in 31 patients. Tissue sections were cut from formalin-fixed, paraffin-embedded blocks containing MM cells and were processed for immunohistochemistry using an automated staining system (BOND-III, Leica Microsystems, Wetzlar, Germany). For assessment of E- and N-cadherin and CD138 expressions, the E-cadherin rabbit monoclonal antibody (Cell Signaling Technology Cat# 3195, RRID:AB_2291471), N-cadherin mouse monoclonal antibody (Abcam Cat# ab98952, RRID:AB_10696943) and CD138 mouse monoclonal antibody (Agilent Cat# M7228, RRID:AB_2254116) were applied at 1:600, 1:4,000 and 1:50 dilutions, respectively, followed by detection using the BOND Polymer Refine Detection kit (Leica Biosystems Cat# DS9800, RRID:AB_2891238). Antigen retrieval was performed with BOND Epitope Retrieval Solution 2 (EDTA, pH=9.0). Positive staining of MM cells was independently evaluated by two pathologists, K.M. and S.H., without any clinical information (Cohen’s κ score 1.0). At a magnification of 200, one field with the highest cadherin positive myeloma cell count on each slide was selected. The percentage of E- or N-cadherin-positive MM cells was calculated using the equation E- or N-cadherin-positive MM cells/total BM nucleated cells, regardless of the number of hematopoietic cells. A score >0.5% was used to determine positivity (yes vs. no) ( Figure 1). The percentage of BM plasma cells was calculated using the equation CD138-positive cells/total BM nucleated cells, regardless of the number of hematopoietic cells.

### Imaging of EMD

The presence of EMD was evaluated by well-trained radiologists. 18F-fluorodeoxyglucose positron emission tomography (PET)/computed tomography (CT) was used in 16/31 cases (51.6%) in total at diagnosis. For the remaining 15 cases, only CT, magnetic resonance imaging (MRI) or both was used in five, four, and six cases, respectively. For the five patients from whom second BM samples were taken during progression, PET/CT, only CT, and both CT and MRI were used in two, two and one cases, respectively. For the patient whose EMD sample was taken during progression, CT was used.

### Statistics

Statistical analysis was performed with SAS 9.4 software (SAS institute, Cary, NC, USA; http://www.sas.com; Statistical Analysis System, RRID:SCR_008567). To compare clinical parameters between cadherin-positive and -negative MM patients, for continuous variables (i.e., age, serum concentrations of albumin, β2 microglobulin, calcium, creatinine, lactate dehydrogenase, hemoglobin, and BM plasma cell %), we performed pooled t-test for equal variances using the t-test analysis. Furthermore, for categorical variables we used the FREQ procedure to perform Fisher’s exact test for the data frequency table (i.e., sex and presence of EMD) because of the large number of cells with count less than five, and chi-square test for monoclonal protein subtypes and the International Staging System. The Kaplan–Meier method was used to analyze the survival probability distribution of the groups accounting for the E-cadherin expression. The statistical significance level was given at *p* <0.05 (two-sided).

## Results

### Relationship between cadherin expression and presence of EMD

Figure 1 shows typical E-cadherin positivity by immunohistochemistry in a patient with MM. Table 1 ^15^ depicts the results of E-cadherin expression in conjunction with various clinical parameters at diagnosis. E-cadherin expression was positive in 25 (80.6%) of the initial 31 BM samples. At initial screening, EMD was found in 14 (45.2%) cases. Four additional patients developed EMD on follow-up from 17 patients who had no EMD at diagnosis. Negative E-cadherin expression at diagnosis (found in six patients) was significantly associated with the presence of EMD ( *p*=0.0041). At diagnosis, all 14 (100%) of the patients with EMD had osseous lesions; none (0%) had extraosseous lesions ( Table 2). No association was found between N-cadherin expression and the presence of EMD.

**Table 1.** Relationship between E-cadherin expression and clinicopathological parameters at diagnosis.

Clinicopathological parameters	E-cadherin Negative ( *n*=6)	E-cadherin Positive ( *n*=25)	*p* value
Age, years (range)	67 (55-82)	67 (46-88)	0.8862
Sex ^#^			1.0000
Male	4	14	
Female	2	11	
Subtype ^♭^			0.4655
IgG	4	12	
IgA	2	8	
Light chain disease	0	5	
Light chain ^#^			0.1719
κ chain	1	14	
λ chain	5	11	
ISS ^♭^			0.3232
I	0	5	
II	5	13	
III	1	7	
Median Alb, g/dl (range)	2.9 (2.2-3.7)	3.6 (2-4.9)	0.0221 *
Median β2MG, mg/l (range)	3.4 (2-5.5)	4.7 (2-15.3)	0.3067
Median Ca, mg/dl (range)	9.9 (8.1-11.8)	9.6 (7.4-12)	0.5797
Median Cr, mg/dl (range)	1.00 (0.7-1.4)	1.18 (0.5-2.89)	0.4588
Median LDH, U/l (range)	190 (132-295)	186 (99-370)	0.8936
Median Hb, g/dl (range)	8.4 (6.8-10)	10.8 (6.5-17.7)	0.0268 *
Median BM PC, % (range)	66.6 (40-90)	62 (10-90)	0.6893
EMD ^#^			0.0041 *
Present	6	8	
Absent	0	17	

ISS, International Staging System; Alb, albumin; β2MG, β2 microglobulin; Ca, calcium; Cr, creatinine; LDH, lactate dehydrogenase; Hb, hemoglobin; BM, bone marrow; PC, plasma cell; EMD, extramedullary disease; IgG, immunoglobulin G; IgA, immunoglobulin A.

^*^ Statistically significant.

^#^ Fisher’s exact test.

^♭^ Chi-square test.

**Table 2.** The EMD sites involved and the number of EMD sites per patient at initial diagnosis.

Patient	EMD sites	EMD number
1	sacral bone, rib bones	6
8	thoracic spine, vertebral canal	2
11	iliac bone	1
13	thoracic spine, rib bone	2
17	humerus bone, rib bones	4
18	thoracic spine	1
22	thoracic spine	2
23	lumbar spine	1
24	thoracic spine, lumbar spine	2
25	lumbar spine	1
26	iliac bone	1
27	iliac bone, cervical spine, rib bone	3
28	sacral bone, iliac bone, lumber spine	5
30	rib bone	1

EMD, extramedullary disease.

In five (patients #25, 28, 29, 30 and 31) of the 31 newly diagnosed patients, four of whom (#25, 28, 29 and 31) were initially positive for E-cadherin and three of whom (#25, 28 and 30) had EMD, we were able to investigate E-cadherin status in an additional BM sample, later in disease progression. In two patients (#25 and 28), the second BM sample became negative for E-cadherin after the first had been positive; in both cases, new EMDs (two additional osseous EMDs in #25 and pleural and chest wall EMDs in #28) were present at the time of the second sample. The two (patients #29 and 31) who were positive for E-cadherin at both biopsies did not show EMD, but patient #30 who was negative for E-cadherin at both biopsies showed osseous EMD throughout the course of the disease, with an increase from one to more than 20. Furthermore, in patient #24, who developed testicular EMD during disease progression, E-cadherin expression was negative both in the BM sample at diagnosis and in the EMD sample at the time of disease progression.

### Relationship between cadherin expression and clinicopathological parameters

**Figure 2.**
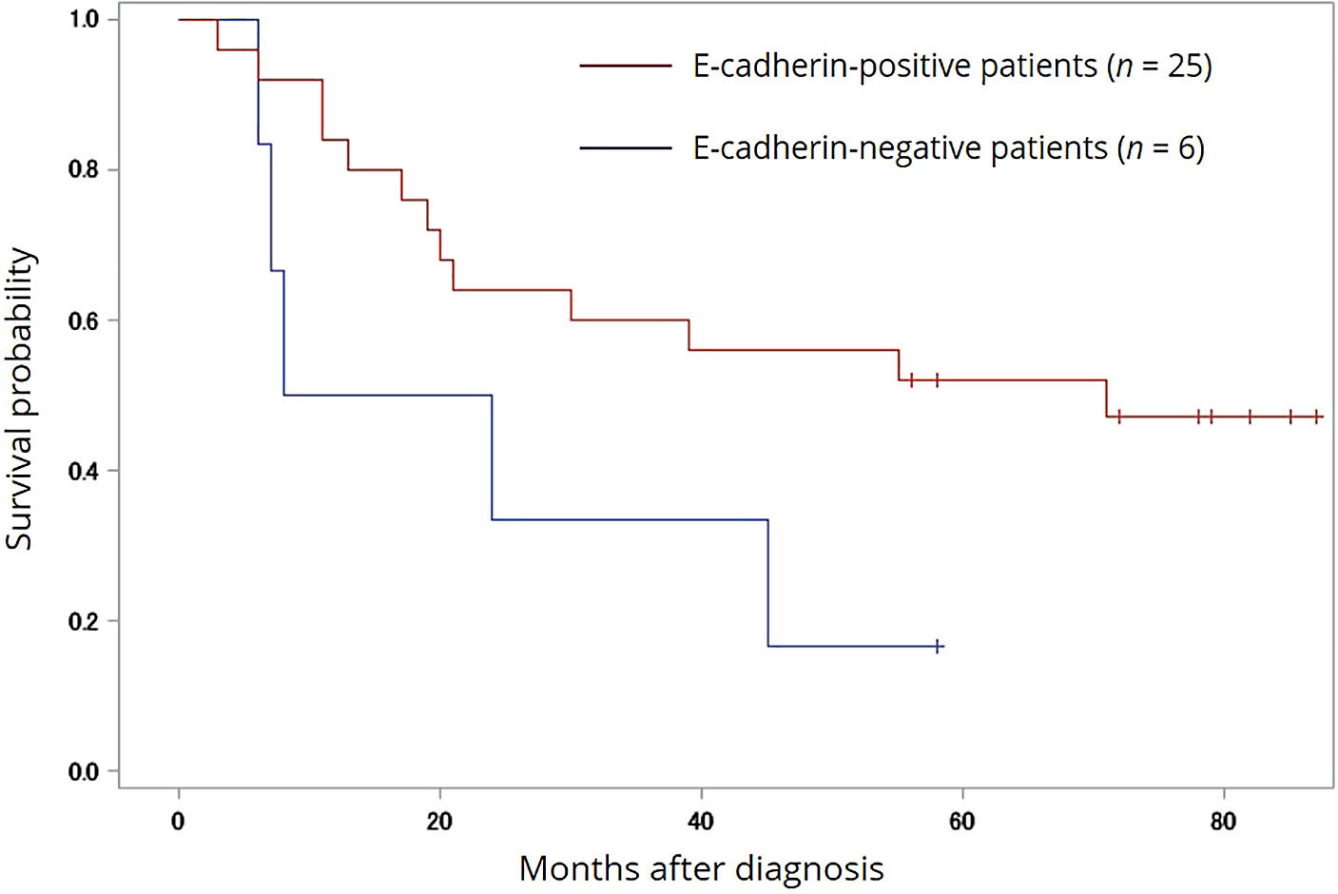
Survival analysis of E-cadherin in univariate analysis. OS stratified by the E-cadherin negativity of MM cells. A statistical significance in OS was found between the E-cadherin-positive and -negative groups (Log-rank *p*=0.0644, Wilcoxon *p*=0.0682, -2Log (LR) *p*=0.0443). Hashmarks indicate surviving patients at last visit. OS, overall survival; MM, multiple myeloma.

Patients with E-cadherin-positive cells had significantly higher serum albumin concentrations ( *p*=0.0221), and hemoglobin values ( *p*=0.0268) ( Table 1). Figure 2 shows the overall survival (OS) probability in all enrolled patients. The median OS values of positive and negative E-cadherin patients were 51.5 months (range 3-93) and 25.8 months (range 6-62), respectively (Log-rank *p*=0.0644, Wilcoxon *p*=0.0682, -2Log (LR) *p*=0.0443). By contrast, N-cadherin expression was not significantly associated with any clinical parameters.

## Discussion

Our present report suggests that the loss of E-cadherin expression in MM cell membranes in BM is involved in the pathogenesis of osseous EMD. Negative E-cadherin expression was significantly associated with the presence of osseous EMD ( *p*=0.0041) ( Table 1). Few studies so far have specifically addressed whether epithelial-to-mesenchymal-transition-like features predict EMD. One experimental study demonstrated that cell lines overexpressing CXCR4 showed decreased E-cadherin. ^2^ This study demonstrated progression to EMD in a mouse model subjected to the injection of this cell line. One clinical study provided evidence that the expression frequency of Twist1, which suppresses E-cadherin, in EMD samples from MM patients, is higher than that in BM samples from these patients or MM patients without EMD. ^5^ In another study, the process of EMD formation has been associated with the upregulation of CXCR4 in EMD specimens of patients with MM. ^6^ Notably, our study is the first to examine the E-cadherin expression of MM cells using BM biopsy specimens from patients.

In agreement with the hypothesis that loss of E-cadherin indicates development of EMD, in two out of five patients with follow-up BM samples (#25 and #28), E-cadherin was found to be positive at the time of diagnosis but negative at the time of disease progression. Interestingly, at progression, the number of osseous EMDs increased in patient #25 and new extraosseous pleural and chest wall EMDs developed in patient #28. In other cases, E-cadherin positivity (#29 and 31) or negativity (#30) was observed in both the first sample and that taken later in the course of the disease; one of these (#30) had EMD. These findings are consistent with the association of negative E-cadherin expression with the higher incidence of EMD, which seemed to be related to the shorter OS. In another patient with sequential samples, #24, a testicular EMD sample during disease progression was investigated. E-cadherin was found to be unexpressed in both the BM at diagnosis and in the EMD at the time of disease progression, possibly indicating the outgrowth of clones without E-cadherin during disease evolution. Taken together, these results suggest that clones without E-cadherin expression in the BM are “fit” under pressure and are assumed to be able to produce specific tropic growth to the EMD site.

Loss of cadherin E expression at diagnosis was observed only in the 43% of the EMD observed cases (6 out of 14). MM is a clinically as well as biologically heterogeneous disease. Thus, we think loss of E-cadherin expression is not only the mechanism for EMD formation, but diverse molecular mechanisms exist for it.

The incidence of osseous EMD in this study was 45.2%. In the literature, osseous EMD is only present in up to 32.5% of newly diagnosed MM cases. ^7^ The higher frequency in our patients is probably explained by the fact that our cohort was enriched by patients using PET/CT (51.6%) at initial staging. Indeed, PET/CT has shown superior potential to regular CT in detecting osseous EMD. ^8^

Another objective of the current study was to investigate whether expression of E-cadherin was associated with clinicopathological parameters, and particularly OS, in these patients. In addition to the significant difference in OS (25.8 vs. 51.5 months; *p*=0.0443), it is noteworthy that negative E-cadherin expression in MM patients in this study was associated with lower serum albumin and hemoglobin levels, suggesting that the loss of this molecule is either a deteriorating factor or a marker for disease severity and shorter OS. Several pieces of circumstantial evidence support the loss of E-cadherin being closely associated with the progression of MM. A study using the MM mouse model showed that decreased E-cadherin expression was associated with tumor progression. ^3^ In a clinical study using DNA-methylation analysis, the E-cadherin gene was shown to be silenced with increased frequency in MM than in patients with monoclonal gammopathies . ^9^ Another clinical study using immunohistochemistry in spinal plasmacytoma, including 28 MMs with osseous EMD and 13 solitary plasmacytomas of bone, showed that patients with negative E-cadherin expression had a significantly shorter OS than those with positive (26.0 vs. 48.7 months; *p* <0.05). ^10^ Although the immunohistochemistry samples were taken from surgically removed spinal masses in their study, their finding was in good accordance with our results in which the immunohistochemistry samples were taken from BM. However, the role of E-cadherin in disease progression and severity is still a matter for debate because the research results are not always consistent. ^11^ As to the relationship between N-cadherin and patient prognosis, Vandyke *et al*. ^12^ revealed an inverse relationship between circulating N-cadherin and patient OS. Contrarily, in our study, no statistically significant relationship was observed between N-cadherin expression in MM cells and patient prognosis.

There are some limitations in this study. The relatively small number of patients and incomplete data on cytogenetics and molecular biology make it difficult for us to assess the direct impact of E-cadherin expression of MM cells on OS. Therefore, further studies with a larger sample size and more consistent follow-up are required to confirm the association of negative E-cadherin expression with poor survival in MM patients with EMD. From the therapeutic viewpoint, preventive strategies to inhibit EMD have been proposed. ^13^^,^^14^ Given the aggressive nature of EMD, biomarkers for patients at risk of EMD will help develop precision medicine for patients with MM.

Taken together, our results suggest that further investigation into the usefulness of E-cadherin expression in simple and routine BM as a biomarker for EMD is warranted.

## Data availability

### Underlying data

Dryad: Negative E-cadherin expression on bone marrow myeloma cell membranes is associated with extramedullary disease. https://doi.org/10.5061/dryad.ns1rn8pvn. ^15^

Data are available under the terms of the Creative Commons Zero “No rights reserved” data waiver (CC0 1.0 Public domain dedication).
