# Supplementary material for: Negative E-cadherin expression on bone marrow myeloma cell membranes is associated with extramedullary disease
Source: F1000Res. 2022 Nov 30;11:245. Originally published 2022 Feb 28. [Version 2] doi: 10.12688/f1000research.109551.2 (PMC9117942; doi:10.12688/f1000research.109551.2)
Supplement: Peer review history

# Peer review: Reviewer response for version 1

**DOI:** 10.5256/f1000research.121064.r134806

**Published:** 2022-05-18

**Authors:** Makoto Sasaki

**Affiliations:** 1 Department of Hematology, Juntendo University School of Medicine, Tokyo, Japan

The authors analyzed E-cadherin expression of clonal plasma cells in bone marrow using the immunohistochemistry staining and revealed the significant association between lack of expression of E-cadherin and osseous extramedullary disease (EMD) in patients with multiple myeloma (MM). Furthermore, they exposed the association of negative expression of E-cadherin with poor survival in patients with MM. Although the sample size is relatively small, this study provides essential information for physician-scientists in the hematological field.

1. There is some evidence of the association between CXCR4 and EMD. In some mouse myeloma models, low expression of CXCR4 is associated with progression of EMD ^1^, and CXCR4 overexpression enhances epithelial-to-mesenchymal transition (EMT) like features with upregulation of Snail, Slug, and Twist, and downregulation of E-cadherin ^2^. It would be more informative if the authors could evaluate and discuss the expression of CXCR4 in their patients.
2. In this report, EMD was observed in 14(45%) of 31 cases at diagnosis. Although, the incidence of EMD in myeloma is around 5% at diagnosis and 20-30% in relapsed/refractory patients ^3^^,^^4^.What is the cause of the extremely high prevalence of EMD in this study group?
3. It is better to add each patient's type of light chain in table 2. In the previous report cited by the authors, the expression of E-cadherin had a strong association with the type of light chain ^5^

Is the work clearly and accurately presented and does it cite the current literature?

Yes

If applicable, is the statistical analysis and its interpretation appropriate?

Yes

Are all the source data underlying the results available to ensure full reproducibility?

Yes

Is the study design appropriate and is the work technically sound?

Yes

Are the conclusions drawn adequately supported by the results?

Yes

Are sufficient details of methods and analysis provided to allow replication by others?

Yes

Reviewer Expertise:

Multiple Myeloma, Clinical Trials

I confirm that I have read this submission and believe that I have an appropriate level of expertise to confirm that it is of an acceptable scientific standard.

## References

[1] : Reduced CXCR4 expression is associated with extramedullary disease in a mouse model of myeloma and predicts poor survival in multiple myeloma patients treated with bortezomib. *Leukemia* .2013;27(10) : 10.1038/leu.2013.148 2075-2077 10.1038/leu.2013.148 23728080PMC4053943

[2] : CXCR4 Regulates Extra-Medullary Myeloma through Epithelial-Mesenchymal-Transition-like Transcriptional Activation. *Cell Rep* .2015;12(4) : 10.1016/j.celrep.2015.06.059 622-35 10.1016/j.celrep.2015.06.059 26190113PMC4961259

[3] : Extramedullary Disease in Multiple Myeloma. *Curr Hematol Malig Rep* .15(2) : 10.1007/s11899-020-00568-3 62-71 10.1007/s11899-020-00568-3 32198576

[4] : How I treat extramedullary myeloma. *Blood* .2016;127(8) : 10.1182/blood-2015-07-635383 971-6 10.1182/blood-2015-07-635383 26679866

[5] : E-cadherin expression on multiple myeloma cells activates tumor-promoting properties in plasmacytoid DCs. *Journal of Clinical Investigation* .2018;128(11) : 10.1172/JCI121421 4821-4831 10.1172/JCI121421 PMC620539630277474

# Peer review: Reviewer response for version 1

**DOI:** 10.5256/f1000research.121064.r126959

**Published:** 2022-04-19

**Authors:** Antonio Bedalov

**Affiliations:** 1 Clinical Research Division, Fred Hutchinson Cancer Research Center, Seattle, WA, USA

Epithelial to mesenchymal transition (EMT) and resulting alterations in cell adhesion, cell polarity, and cytoskeleton remodeling have been associated with the acquisition of invasive and metastatic properties of cancer cells. EMT is orchestrated by a well-characterized set of transcription factors (e.g. SNAIL, TWIST) which change the expression of several adhesion proteins, including downregulation of E-cadherin and upregulation of N-cadherin. Even though the hematopoietic system is mesoderm-derived, an EMT-like transcriptional cascade has been described in hematologic cancers, including multiple myeloma (MM) as shown in a mouse model of MM progression ^1^.

 A major finding of the study is that the lack of expression of E-cadherin in diagnostic bone marrow biopsy of 31 multiple myeloma patients is associated with the presence of extramedullary disease. All 6 patients that lack E-cadherin expression exhibit extramedullary disease (EMD) 6/6=100%), while among the 25 patients that express E-cadherin EMD is found at diagnosis in only 8 patients (8/25=32%). Even though the sample size is small, this association is significant and strong, which strengthens the argument that the EMT might be involved in the acquisition of invasive phenotype in MM. The authors have also observed that the absence of cadherin E expression is associated with reduced survival, however, it is presently not clear whether the lack of cadherin E expression was an independent prognostic marker or just a marker of more severe/advanced disease, given that other markers of disease severity such as reduced albumin and hemoglobin level are also associated with the absence of cadherin E expression. While limitations of the studies are mentioned. I believe that additional points that should be brought up in the discussion are:

1. Less than half of the patients with EMD exhibit loss of cadherin E expression at diagnosis (does this reflect MM heterogeneity, sampling error, or do the authors believe that invasive phenotype occurs in EMT-independent fashion);
2. Does the prognosis of the patients with EMD but without loss of cadherin E differ from that in patients with EMD and loss of cadherin E;
3. Are there patients for which the biopsy of both EMD and bone marrow biopsy have been obtained, and if so, what was the concordance in cadherin E expression between the two?

If unable to address these points directly because of the small sample size, this limitation should be acknowledged and discussed.

Is the work clearly and accurately presented and does it cite the current literature?

Yes

If applicable, is the statistical analysis and its interpretation appropriate?

Yes

Are all the source data underlying the results available to ensure full reproducibility?

Yes

Is the study design appropriate and is the work technically sound?

Yes

Are the conclusions drawn adequately supported by the results?

Yes

Are sufficient details of methods and analysis provided to allow replication by others?

Yes

Reviewer Expertise:

Cancer biology, hematologic malignancies, epigenetics, DNA replication

I confirm that I have read this submission and believe that I have an appropriate level of expertise to confirm that it is of an acceptable scientific standard.

## References

[1] : Hypoxia promotes dissemination of multiple myeloma through acquisition of epithelial to mesenchymal transition-like features. *Blood* .2012;119(24) : 10.1182/blood-2011-09-380410 5782-94 10.1182/blood-2011-09-380410 22394600PMC3382938

# Response

**Published:** 2022-05-09

**Authors:** Maki Hirao

**Affiliations:** Tokyo Saiseikai Central Hospital, Japan

Thank you for your useful comments and suggestions. The reviewer has asked for a description of why loss of cadherin E expression at diagnosis was observed only in the 43% of the EMD observed cases (6 out of 14). MM is a clinically as well as biologically heterogeneous disease. Thus, we think loss of E-cadherin expression is not only the mechanism for EMD formation, but diverse molecular mechanisms exist for it. Currently, the small number of patients has only been used in assessment of the direct impact of E-cadherin expression of MM cells on OS, and we are unable to rule out the possibility of sampling errors. Therefore, our results require further studies with a larger sample size and more consistent follow-up. We will include this explanation in the discussion of the revised manuscript.

 Additional data analysis was performed to address potential roles of E-cadherin. Among the 14 patients with EMD, no correlation was found between E-cadherin expression and the clinical outcome. The median OS values of positive and negative E-cadherin patients were 34.9 months (range 5-87) and 25.8 months (range 6-62), respectively (Log-rank p=0.8459, Wilcoxon p=0.6507, -2Log (LR) p=0.6663). Further studies should be addressed to investigate the potential role of cadherin E in EMD patients for prognostic prediction.

 We note that the reviewer is concerned about the correlation in cadherin E expression between BM staining and EMD lesion. To address this issue, we have validated the concordance in cadherins E expression between the testicular EMD and BM biopsies only in patient #24. E-cadherin was found to be unexpressed in both the BM and the EMD.  The findings are described on line 27 to 28 of page 4 and line 13 to 16 of page 6 of the manuscript. We are planning to carry out further studies on the EMD biopsies.
